# Systemic Inflammation Indices and Association with Prostate Cancer Survival in a Diverse Patient Cohort

**DOI:** 10.3390/cancers15061869

**Published:** 2023-03-20

**Authors:** Maeve Bailey-Whyte, Tsion Z. Minas, Tiffany H. Dorsey, Cheryl J. Smith, Christopher A. Loffredo, Stefan Ambs

**Affiliations:** 1 School of Medicine, University of Limerick, V94 XD21 Limerick, Ireland; 2 Laboratory of Human Carcinogenesis, Center for Cancer Research, National Cancer Institute (NCI), National Institutes of Health (NIH), Bethesda, MD 20892, USA; 3 Cancer Prevention and Control Program, Lombardi Comprehensive Cancer Center, Georgetown University Medical Center, Washington, DC 20057, USA

**Keywords:** prostate cancer, systemic inflammation indices, African American, health disparity, inflammation

## Abstract

**Simple Summary:**

Systemic inflammation indices are defined by peripheral blood count combinations of cell types such as neutrophils, lymphocytes, and monocytes. Elevated levels of these indices have previously been associated with increased cancer mortalities including prostate cancer. Studies investigating systemic inflammation indices in African American men with prostate cancer are lacking despite the evidence according to which chronic inflammation is a candidate risk factor for the disease among these men. We investigated the association of four systemic inflammation indices with prostate cancer mortality by measuring them from blood counts. Our participants included self-identified African American and European American patients. We found that high levels of these indices were significantly associated with all-cause and prostate cancer-specific mortality among all men combined. Some of these associations were observed for African American men but not European American men in the race/ethnicity stratified survival analysis. These findings suggest that inflammation indices could be predictors of prostate cancer mortality.

**Abstract:**

There is a lack of investigations assessing the performance of systemic inflammation indices as outcome predictive tools in African Americans with prostate cancer. This study aims to assess the relationships between neutrophil-to-lymphocyte ratio (NLR), platelet-to-lymphocyte ratio (PLR), systemic immune-inflammation (SII), and systemic inflammation response index (SIRI) with survival outcomes among 680 diverse men with prostate cancer. Routine blood results were collected from self-identified African American and European American patients. We applied multivariable Cox regression modeling to examine the associations of systemic inflammation indices with overall and prostate cancer-specific survival. The median survival follow-up was 5.9 years, with 194 deaths. NLR, SII, and SIRI, but not PLR, showed associations with all-cause and prostate cancer-specific mortality when coded as dichotomized and continuous variables. NLR and SIRI were significantly associated with prostate cancer-specific mortality among all men (hazard ratio (HR) 2.56 for high vs. low NLR; HR 3.24 for high vs. low SIRI) and African American men (HR 2.96 for high vs. low NLR; HR 3.19 for high vs. low SIRI). Among European Americans, only SII showed an association with prostate cancer-specific survival. These observations suggest that inflammation indices merit further study as predictors of prostate cancer mortality.

## 1. Introduction

Men of African descent have an increased rate of metastatic and fatal prostate cancer compared to other men [1,2]. Equal access to care remains an essential goal to improve this health disparity. However, there are also well-established differences in disease biology across population groups that may contribute to these poorer outcomes. It is hypothesized that systemic inflammation contributes to the prostate cancer disparities in the United States. Notable differences in the activation of immune–inflammatory pathways and inflammatory mediators have been reported between African American (AA) and European American (EA) men with prostate cancer [3]. Additionally, an immune and inflammation signature consistent with the activation of inflammation and immune-modulatory pathways has been detected with greater prevalence in prostate tumors from African American men compared to EA men [4,5]. The upregulation of genes in the interferon pathway forms part of this signature and its presence has been associated with a reduction in disease-free survival in men with prostate cancer [5]. Regular use of aspirin is associated with decreased odds of lethal prostate cancer in AA men [6,7,8]. Collectively, this evidence suggests that low-grade chronic inflammation may be a predictive factor for adverse outcomes in AA prostate cancer patients.

Systemic inflammation (SI) indices are a method of quantifying systemic inflammation. Four commonly used indices in the scientific literature are neutrophil-to-lymphocyte ratio (NLR), platelet-to-lymphocyte ratio (PLR), systemic immune-inflammation (SII), and systemic inflammation response index (SIRI). They have shown promise as predictive indicators of disease outcomes across multiple cancer types including breast cancer [9,10,11]. SI indices are cost-effective and easily accessible through standard blood analyses, and therefore represent a viable option for clinical practice worldwide. In prostate cancer, multiple studies have already shown that some of these indices are predictive of survival in localized [12,13] and metastatic prostate cancer [14,15,16]. However, there is a lack of studies assessing how these ratios perform as predictive tools across population subgroups. In colon cancer, elevated platelets have been associated with increased risk of death in AA men [17]. In prostate cancer, men of African descent are at higher risk of aggressive disease and may also have increased systemic inflammation, but the value of these systemic inflammation indices in this group is unknown.

This study will assess the relationship between NLR, PLR, SII, and SIRI and survival outcomes in a diverse population of men with prostate cancer.

## 2. Materials and Methods

### 2.1. Study Population

The study population from the National Cancer Institute (NCI)-Maryland prostate cancer case–control study was used for this investigation. The NCI-Maryland study was initiated in 2005 and recruitment ended in 2015. The study was undertaken to test the primary hypothesis according to which environmental exposures and ancestry-related factors contribute to the disparities in prostate cancer burden experienced by AA men [6,18]. Before being interviewed, all individuals signed informed consent for participation. All study forms and procedures were approved by the NCI (protocol # 05-C-N021) and the University of Maryland’s (protocol #0298229) institutional review boards. Research followed the ethical guidelines set by the Declaration of Helsinki. Cases were recruited at the Baltimore Veterans Affairs Medical Center and the University of Maryland Medical Center and controls were identified through the Maryland Department of Motor Vehicle Administration database. Controls were frequency-matched to cases on age and race. This article follows the Strengthening the Reporting of Observational Studies in Epidemiology (STROBE) guidelines for the reporting of observational studies.

### 2.2. Data Collection

This is a case-only analysis. All enrolled cases had a prostate cancer diagnosis within 2 years of recruitment into the study. The clinical and pathological parameters of patients were collected through chart review and questionnaire. Race/ethnicity was self-reported as either black or AA or as white or EA as part of the eligibility screener and within the questionnaire. Routine blood results were also collected, including total white blood cell count, neutrophil count, monocyte count, lymphocyte count, and platelet count.

### 2.3. Statistical Analysis

Statistical analysis was conducted using the Stata/SE 17.0 statistical software package (StataCorp, College Station, TX, USA). All statistical tests were 2-sided. An association was considered statistically significant with *p*  <  0.05. NLR, PLR, SII, and SIRI were calculated as follows: NLR = neutrophil count/lymphocyte count; PLR = platelet count/lymphocyte count, SII = platelet count × neutrophil count/lymphocyte count, and SIRI = neutrophil count × monocyte count/lymphocyte count. ROC curves and the Youden Index were used to identify the optimal cut-off values for NLR, PLR, SII, and SIRI with the highest sensitivity and specificity. For analysis, we assessed the systemic inflammation indices as either a continuous measure or categorical variable. Differences between groups were determined using Pearson’s chi-squared test.

For survival analysis with the Kaplan–Meier method, the log-rank test was used to examine differences in all-cause and prostate cancer-specific mortality according to levels of systemic inflammation indices, with indices being dichotomized based on cut-offs determined by receiver operating characteristic curves and the Youden Index [19,20]. Cox regression models were used to estimate adjusted hazard ratios (HRs) and 95% confidence intervals (CIs) for all-cause mortality and prostate cancer-specific mortality in cases. For this analysis, we used both dichotomized and continuous index data. Median duration of survival follow-up was 5.9 years. In the analysis of all-cause mortality, median follow-up time to death from any cause was 5.3 years for AA men and 6.8 years for EA men. In the analysis of prostate cancer-specific survival, median follow-up time to death from prostate cancer was 3.5 years for AA men and 8.2 years for EA men. We adjusted for the following potential confounding factors: age at recruitment, body mass index, diabetes, aspirin use, education, family history of prostate cancer, self-reported race, smoking history, treatment, disease stage, and Gleason score. We calculated survival for cases from date of diagnosis to either date of death or to the censor date of 31 December 2020. We confirmed non-violation of the proportionality assumption based on the goodness-of-fit test using Schoenfeld residuals. We used log-rank test of equality of survivor functions to assess statistically significant heterogeneity between hazard ratios.

## 3. Results

### 3.1. ROC Analysis of Systemic Inflammation Indices

Optimal cut offs for the systemic indices as assessed by ROC curves and Youden Index were 2.9 for NLR, 133.7 for PLR, 430.8 for SII, and 0.9 for SIRI. For each systemic inflammation index, patients were divided into two groups for further analysis; NLR ≤ 2.9 (low) and NLR > 2.9 (high); PLR ≤ 430.8 (low) and PLR > 133.7 (high); SII ≤ 340 (low) and SII > 340 (high); SIRI ≤ 0.9 (low) and SIRI > 0.9 (high). The results of the ROC curve analysis are presented in Supplementary Figure S1 and Supplementary Table S1.

### 3.2. Clinical and Demographic Characteristics of Participants

The NCI-Maryland prostate cancer study enrolled 976 cases (489 AA men and 487 EA men), as part of a case–control design [5,17], and additional cases not being part of the case–control study. For this study, we included all cases with available blood counts. Cases with a complete blood count measure taken 1 year or more after biopsy were excluded (*n* = 61). Additionally, blood count measures were not available for all cases in the study (Supplementary Table S2). The final numbers of participants available for analysis were *n* = 680 for NLR, *n* = 679 for PLR and SII, and *n* = 678 for SIRI. 

The demographic and disease characteristics of the eligible subjects are shown in Table 1, stratified by the dichotomized systemic inflammation indices. Differences between AA and EA cases were assessed. A smaller proportion of EA men were current smokers compared to AA men. EA men also had higher levels of educational attainment compared to AA men. AA men with low NLR had a higher proportion of stage 4 cases compared to EA men.

We also assessed differences between the low and high SI Indices groups within population groups. EA men with elevated NLR were statistically older than EA men with low NLR but there was no difference in age for AA men across the NLR groups. There was no difference in age between men with high or low PLR or SII. EA men with elevated SIRI were older than men with low SIRI. There was no difference in BMI between men when stratified into low and high NLR and SII. AA men with low PLR had a higher BMI compared to AA men with high PLR. EA men with high SIRI had a higher BMI. 

Median PSA was significantly different for both AA and EA men when stratified into low and high NLR. Median PSA differed for AA men across PLR and SIRI groups with no differences across groups for SII. EA men with high NLR were more likely to have stage 4 disease compared to EA men with low NLR. We report no differences in the educational attainment of AA or EA men, smoking status, family history of prostate cancer, Gleason score, and disease aggressiveness when stratified into low and high SI indices.

### 3.3. Systemic Inflammation Indices and All-Cause Mortality in Men with Prostate Cancer

**Figure 1:**
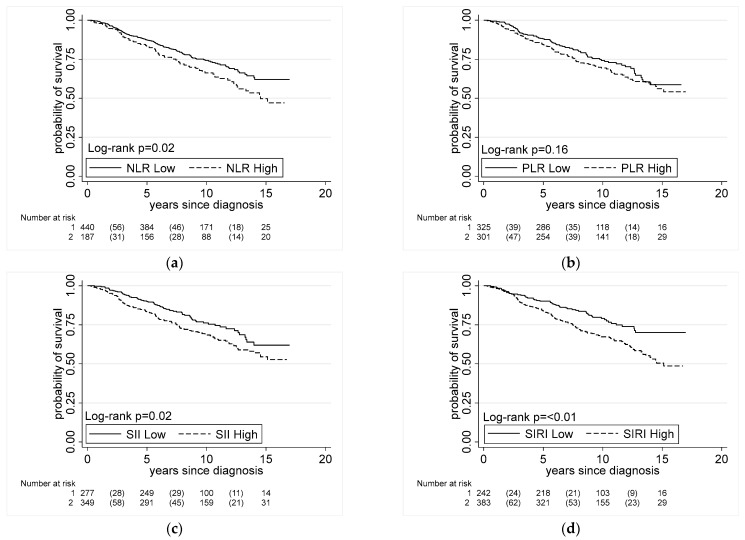
Association between all-cause mortality and systemic inflammation indices. Kaplan–Meier survival plots showing association of systemic inflammation indices and all-cause mortality in men with prostate cancer. Associations in all cases for (**a**) NLR (low ≤ 2.9, high > 2.9), (**b**) PLR (low ≤ 133.7, high > 133.7), (**c**) SII (low ≤ 430.8, high > 430.8), and (**d**) SIRI (low ≤ 0.9, high > 0.9).

Firstly, we assessed whether there was an association between elevated systemic inflammation indices and all-cause mortality in men with prostate cancer. Up to the end of 2020, 194 men in our study had died (*n* = 114 AA, *n* = 80 EA). PLR was not found to be associated with all-cause mortality. In Kaplan–Meier unadjusted analyses, we identified associations between NLR, SII, and SIRI and all-cause mortality (Figure 1). In the multi-variable Cox regression analysis, there were positive associations between NLR (HR = 1.23, 95% CI = 1.03 to 1.48), SII (HR = 1.20, 95% CI = 1.02 to 1.41), and SIRI (HR = 1.17, 95% CI = 1.02 to 1.33) when data were analyzed as continuous variables (Table 2). These positive associations remained for SII (HR = 1.47, 95% CI = 1.04 to 2.09) and SIRI (HR = 1.65, 95% CI = 1.14 to 2.41) when the variables were dichotomized. 

We previously reported that AA men in the NCI-Maryland study were generally more likely to die after a prostate cancer diagnosis than EA men, but they were also more likely to die from prostate cancer [7]. In order to investigate whether the two patient groups are differently affected by systemic inflammation, we stratified the data by self-reported race into AA and EA. Generally, we observed similar trends for the association of these indices with all-cause mortality among the AA and EA patients. However, none of those associations reached statistical significance for EA patients. Among the AA patients, there was an association of SII with all-cause mortality in the multi-variable Cox regression analysis, with a significant association for high vs. low SII (HR = 1.66, 95% CI = 1.06 to 2.60), and with SIRI as a continuous variable (HR = 1.22, 95% CI = 1.02 to 1.46); the latter association approached statistical significance in the dichotomized approach (HR = 1.55, 95% CI = 0.99 to 2.43) (Table 2).

### 3.4. Systemic Inflammation Indices and Prostate Cancer-Specific Mortality

**Figure 2:**
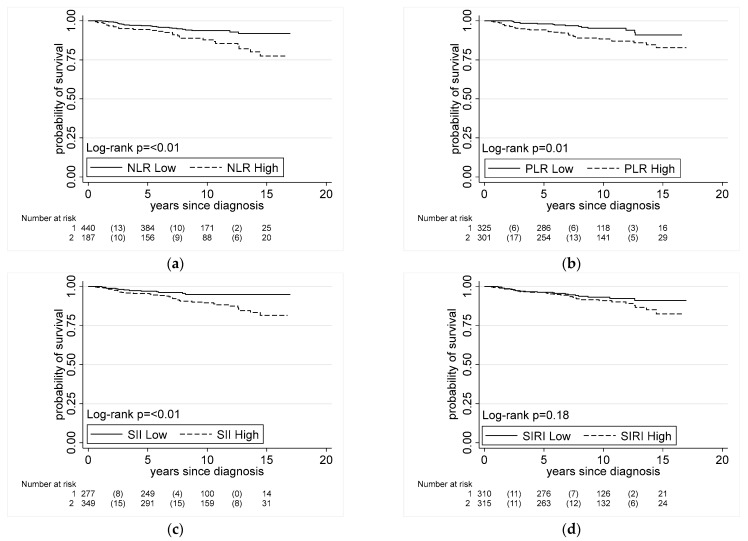
Association between prostate cancer-specific mortality and systemic inflammation indices. Kaplan–Meier survival plots showing association of systemic inflammation indices and prostate cancer-specific mortality in men. Associations in all cases for (**a**) NLR (low ≤ 2.9, high > 2.9), (**b**) PLR (low ≤ 133.7, high > 133.7), (**c**) SII (low ≤ 430.8, high > 430.8), and (**d**) SIRI (low ≤ 0.9, high > 0.9).).

We investigated the association between systemic inflammation indices and prostate cancer-specific mortality in our case population. By the end of 2020, 50 of the cases in our dataset had died of prostate cancer (*n* = 33 AA, *n* = 17 EA). High NLR, PLR, and SII were all associated with prostate cancer-specific mortality in the unadjusted analysis (Figure 2). In the fully adjusted model, NLR, SII, and SIRI remained associated with prostate cancer-specific mortality among all men using dichotomized and continuous exposure data in our models, although the confidence intervals were rather wide for the dichotomized analysis (Table 3). To further understand the importance of this finding for AA men, we stratified the analysis by race/ethnicity. Here, we found that NLR and SIRI were associated with prostate cancer-specific mortality among AA men, e.g., HR 2.96, 95% CI = 1.1 to 7.98 for high vs. low NLR; HR 3.19, 95% CI = 1.12 to 9.04 for high vs. low SIRI. Among European Americans, only dichotomized SII showed a significant association with prostate cancer-specific survival (HR 11.63, 95% CI = 1.03 to 131 for high vs. low SII), but with a wide confidence interval. Yet, as a note of caution, in the race/ethnicity-stratified survival analysis with dichotomized index data, generally few deaths occurred among men in the low index group, making the HR estimates somewhat uncertain.

## 4. Discussion

In this study, we report that three commonly used systemic inflammation indices, NLR, SII, and SIRI, were associated with all-cause mortality among men with prostate cancer in our study, particularly for AA men. Our findings add to accumulating evidence for the role of systemic inflammation indices as prognostic tools for risk stratification and treatment choices. This study also supports prior evidence for systemic inflammation as a risk factor for prostate cancer progression in men of African descent.

Previous studies indicate a role for systemic inflammation indices as prognostic tools of advanced or lethal prostate cancer. SII was reported as a prognostic factor for overall survival in men with metastatic castration-resistant prostate cancer (mCRPC) treated with first-line docetaxel [21]. In men with mCRPC treated with abiraterone after docetaxel, SII and NLR could also predict overall survival [14]. In pancreatic cancer, the ability of SIRI to predict survival outperformed NLR, and patients who had high SIRI scores showed increased serum concentrations of inflammatory cytokines, suggesting that SIRI can reflect the status of both the local immune response and systemic inflammation [22].

Our findings, in agreement with the previous literature, show an association between NLR, SII, and SIRI and all-cause mortality in men with prostate cancer. Importantly, our diverse patient population allowed us to further assess this association in AA and EA men separately. This novel study revealed a more robust association for SII and SIRI in AA men, suggesting a role for these peripheral blood count tools in a population subgroup which is at higher risk of adverse prostate cancer outcomes.

SII and SIRI represent a combination of three parameters from peripheral blood counts, and so it has been suggested that they offer superior accuracy as predictive tools when compared to NLR and PLR in urinary system cancers [16,23]. The exact mechanism for these systemic inflammation indices being prognostic tools remains unclear but is attributed to the pathophysiological role of the peripheral blood to systemic inflammatory responses and cancer progression. Neutrophils promote tumor initiation and progression through roles in immunosuppression [24], angiogenesis [25], and metastasis [26]. Low lymphocyte counts indicate an insufficient host immune response and are associated with adverse clinical outcomes, perhaps due to a reduced level of CD4+ T cells, which impairs tumor suppression and defense [27]. Platelets play an established role in carcinogenesis [28,29]. Cancer cells engage platelet aggregation and activation to support tumor cell survival and in the establishment of a pre-metastatic niche through cyclooxygenase signaling [30].

Immune cell numbers are known to differ between AA men and EA men. Healthy AA men tend to have lower white blood cell counts and absolute neutrophil counts when compared to EA men [31]. In colorectal cancer, AA men with elevated platelets at diagnosis had a higher risk of death compared to EA men [17]. This difference by ethnicity highlights the nuance required when trying to ascertain the value of biomarkers as prognostic tools across population subgroups and suggests that differences in the immune response may contribute to disparities in survival. The differences are attributed in part to pro-inflammatory lifestyle behaviors such as smoking, chronic infections, and genetic ancestry, but this has not been fully established.

Anti-inflammatories are being investigated as adjuvant therapeutic options to prevent prostate cancer progression. The non-steroidal anti-inflammatory (NSAID) drug mefenamic acid has been investigated as a treatment to decrease biochemical recurrence in men with castration-resistant prostate cancer. The small phase II/III clinical trial (Cuban Public Registry of Clinical Trials Database RPCEC00000248) with ten patients in each arm had a study endpoint of a change in serum PSA at 6 months with biochemical recurrence defined as an increase of ≥25% in PSA levels [32]. In the group treated with mefenamic acid, there was a 42% decrease in serum PSA level when compared to the placebo arm. In addition, 70% of the placebo arm exhibited biochemical disease progression in comparison to none of the patients treated with mefenamic acid. Mechanistically, mefenamic acid is a known inhibitor of the pro-inflammatory NLRP3 inflammasome, which is suggested to be at least partially responsible for the elevated NLR that we see reported across cancer types [33].

Additionally, regular aspirin use has been associated with a significantly reduced risk of both advanced prostate cancer and disease recurrence in AA men [6,34]. Studies in both mice and humans have identified the inhibition of the cyclooxygenase/thromboxane A2 pathway as a potential mechanism of action for aspirin in the prevention of metastatic cancer [8,30]. Importantly, elevated TXB2 (the stable metabolite of TXA2) is associated with adverse survival outcomes for African American men and is inversely associated with aspirin use. This suggests a benefit for aspirin use in preventing lethal prostate cancer in this high-risk group of men through TXA2 inhibition. The building evidence for use of anti-inflammatories as adjuvant treatments in prostate cancer adds support to our findings of a possible predictive role for inflammation markers in prostate cancer.

A key strength of this study is the participant diversity. This study included comparable numbers of AA (57%) and EA cases (44%) linked to a unique dataset that included complete blood count measures and long-term follow up of all cause and disease-specific deaths. Limitations to our study arise from the few deaths related to prostate cancer on follow-up, being limited to 50 among the 680 patients. Due to this relatively small number of prostate cancer deaths, further stratification of our survival analysis by race/ethnicity may have yielded imprecise HR estimates.

## 5. Conclusions

In our study, systemic inflammation indices were found to be related to both all-cause and disease-specific mortality among prostate cancer patients. NLR and SIRI were significantly associated with prostate cancer-specific mortality among all men and AA men. SIRI was associated with both all-cause and disease-specific mortality in AA men. Identifying novel biomarkers of aggressive disease is important for high-risk groups such as men of African ancestry who experience an excessive burden of lethal prostate cancer. Our findings support considering the incorporation of systemic inflammation indices into the clinical decision-making processes for risk stratification and treatment strategies.

## Figures and Tables

**Table 1:** Baseline patient characteristics based on neutrophil–lymphocyte ratio (NLR), platelet–lymphocyte ratio (PLR), systemic immune–inflammation index (SII), and systemic inflammation response index (SIRI).

Cases ^a^	NLR					PLR					SII					SIRI				
		Low		High			Low		High			Low		High			Low		High	
		≤2.9		>2.9			≤133.7		>133.7			≤430.8		>430.8			≤0.9		>0.9	
Demographics of Cases	All ^m^ (*n* = 680)	AA ^b^ (*n* = 305)	EA ^c^ (*n* = 172)	AA (*n* = 86)	EA (*n* = 117)	All (*n* = 679)	AA ^b^ (*n* = 212)	EA ^c^ (*n* = 144)	AA ^c^ (*n* = 178)	EA ^c^ (*n* = 145)	All (*n* = 679)	AA ^b^ (*n* = 203)	EA ^c^ (*n* = 97)	AA ^c^ (*n* = 187)	EA ^c^ (*n* = 192)	All (*n* = 678)	AA ^b^ (*n* = 190)	EA ^c^ (*n* = 70)	AA ^c^ (*n* = 200)	EA ^c^ (*n* = 217)
Age ^d^																				
Median (IQR ^e^) in years	63 (10)	63 (9)	63 (9)	64 (11)	65 (10)	63 (10)	63 (10)	64 (10)	64 (10)	64 (11)	63 (10)	63 (10)	63 (9)	63 (10)	64 (11)	63 (10)	63 (9)	60 (9)	63 (10)	64 (11)
BMI																				
Mean (SD ^f^) in kg/m^2^	28.2 (5.0)	28.3 (5.6)	28.4 (4.1)	27.1 (4.9)	28.2 (4.9)	28.2 (5.0)	28.8 (5.6)	28.8 (4.3)	27.2 (5.3)	27.9 (4.6)	28.2 (5.0)	28.4 (5.2)	28.2 (3.9)	27.7 (5.8)	28.4 (4.7)	28.2 (5.0)	28.0 (5.0)	27.1 (3.3)	28.2 (6.0)	28.7 (4.7)
Education, *N* (%)																				
High school or less	264 (39)	137 (45)	51 (30)	36 (42)	40 (34)	263 (39)	90 (42)	47 (32)	82 (46)	44 (30)	263 (39)	93 (46)	29 (30)	79 (42)	62 (32)	263 (39)	81 (43)	18 (26)	91 (46)	73 (34)
Some college	230 (34)	117 (38)	59 (34)	27 (31)	27 (23)	230 (34)	81 (38)	48 (33)	63 (35)	38 (26)	230 (34)	76 (37)	30 (31)	68 (36)	56 (29)	230 (34)	75 (39)	19 (27)	69 (34)	66 (30)
College	84 (12)	25 (8)	26 (15)	10 (12)	23 (20)	84 (12)	18 (8)	24 (17)	17 (10)	25 (17)	84 (12)	18 (9)	17 (18)	17 (9)	32 (17)	84 (12)	19 (10)	13 (19)	16 (8)	36 (17)
Graduate	47 (7)	11 (4)	15 (9)	4 (5)	17 (15)	47 (7)	9 (4)	11 (8)	6 (3)	21 (14)	47 (7)	7 (3)	6 (6)	8 (4)	26 (14)	47 (7)	5 (3)	8 (11)	10 (5)	24 (11)
Did not provide	55 (8)	15 (5)	21 (12)	9 (10)	10 (8)	55 (8)	14 (7)	14 (10)	10 (6)	17 (12)	55 (8)	9 (4)	15 (15)	15 (8)	16 (8)	54 (8)	10 (5)	12 (17)	14 (7)	18 (8)
Baseline Health Factors																				
Family history of prostate cancer ^g^, *N* (%)																				
No	546 (80)	255 (84)	125 (73)	68 (79)	98 (84)	545 (80)	172 (81)	109 (76)	150 (84)	114 (79)	545 (80)	169 (83)	66 (68)	153 (82)	157 (82)	545 (80)	161 (85)	46 (66)	161 (81)	176 (81)
Yes	78 (11)	33 (11)	25 (14)	9 (10.5)	11 (9)	78 (11)	24 (11)	21 (14)	18 (10)	15 (10)	78 (12)	23 (11)	16 (16)	19 (10)	20 (10)	78 (11)	19 (10)	12 (17)	23 (11)	24 (11)
Did not provide	56 (8)	17 (6)	22 (13)	9 (10.5)	8 (7)	56 (8)	16 (8)	14 (10)	10 (6)	16 (11)	56 (8)	11 (5)	15 (15)	15 (8)	15 (8)	55 (8)	10 (5)	12 (17)	16 (8)	17 (8)
Smoking status ^h^, *N* (%)																				
Current	190 (28)	106 (35)	33 (19)	30 (35)	21 (18)	189 (28)	77 (36)	32 (22)	58 (33)	22 (15)	189 (28)	66 (32)	16 (16)	69 (37)	38 (20)	190 (28)	67 (35)	12 (17)	69 (34)	42 (19)
Former	254 (37)	100 (33)	68 (40)	28 (33)	58 (50)	254 (37)	67 (32)	61 (42)	61 (34)	65 (45)	254 (37)	67 (33)	42 (43)	61 (33)	84 (44)	254 (37)	62 (33)	29 (41)	66 (33)	96 (44)
Never	182 (27)	83 (27)	52 (30)	19 (22)	28 (24)	182 (27)	54 (25)	39 (27)	48 (27)	41 (28)	182 (27)	60 (30)	26 (27)	42 (22)	54 (28)	181 (27)	50 (26)	18 (26)	51 (26)	62 (29)
Did not provide	54 (8)	16 (5)	19 (11)	9 (10)	10 (8)	54 (8)	14 (7)	12 (8)	11 (6)	17 (12)	54 (8)	10 (5)	13 (13)	15 (8)	16 (8)	53 (8)	11 (6)	11 (16)	14 (7)	17 (8)
Stage ^i^, *N* (%)																				
T1	117 (17)	50 (16)	37 (22)	14 (16)	16 (14)	117 (17)	39 (18)	30 (21)	25 (14)	23 (16)	117 (17)	37 (18)	22 (23)	27 (14)	31 (16)	116 (17)	35 (18)	20 (28)	29 (15)	32 (15)
T2	461 (68)	220 (72)	108 (63)	54 (63)	79 (68)	460 (68)	147 (69)	92 (64)	126 (71)	95 (65)	460 (68)	144 (71)	57 (59)	129 (69)	130 (68)	461 (68)	131 (69)	41 (59)	143 (71)	145 (67)
T3	54 (8)	16 (5)	22 (13)	7 (8)	9 (8)	54 (8)	17 (8)	17 (12)	6 (3)	14 (10)	54 (8)	12 (6)	13 (13)	11 (6)	18 (9)	54 (8)	15 (8)	7 (10)	8 (4)	24 (11)
T4	44 (6)	17 (6)	4 (2)	10 (12)	13 (11)	44 (6)	6 (3)	4 (3)	21 (12)	13 (9)	44 (6)	9 (4)	4 (4)	18 (10)	13 (7)	43 (6)	8 (4)	2 (3)	18 (9)	15 (7)
Missing	4 (1)	2 (1)	1 (<1)	1 (1)		4 (1)	3 (1)	1 (1)			4 (1)	1 (<1)	1 (1)	2 (1)		4 (1)	1 (1)		2 (1)	1 (<1)
Gleason score, *N* (%)																				
≤7	549 (81)	251 (82)	142 (83)	64 (74)	92 (79)	548 (81)	175 (83)	116 (81)	139 (78)	118 (81)	548 (81)	171 (84)	77 (79)	143 (76)	157 (82)	548 (81)	158 (83)	62 (89)	157 (78)	170 (78)
>7	126 (18)	51 (17)	30 (17)	20 (23)	25 (21)	126 (18)	33 (16)	28 (19)	38 (21)	27 (19)	126 (18)	31 (15)	20 (21)	40 (21)	35 (18)	125 (18)	30 (16)	8 (11)	40 (20)	47 (22)
Missing	5 (1)	3 (1)		2 (2)		5 (1)	2 (2)		1 (1)		5 (1)	1 (1)		4 (2)		5 (1)	2 (1)		3 (2)	
Disease aggressiveness, *N* (%)																				
Nonaggressive disease ^j^	491 (72)	230 (75)	125 (73)	58 (68)	78 (67)	490 (72)	159 (75)	104 (72)	128 (72)	99 (68)	490 (72)	158 (78)	67 (69)	129 (69)	136 (71)	490 (72)	144 (76)	55 (79)	144 (72)	146 (67)
Aggressive disease ^k^	185 (27)	73 (24)	46 (27)	27 (31)	39 (33)	185 (27)	50 (24)	39 (27)	50 (28)	46 (32)	185 (27)	44 (22)	29 (30)	56 (30)	56 (29)	184 (27)	45 (24)	15 (21)	54 (27)	70 (32)
Missing	4 (1)	2 (1)	1 (<1)	1 (1)		4 (1)	3 (1)	1 (1)			4 (1)	1 (1)	1 (1)	2 (1)		4 (1)	1 (1)		2 (1)	1 (1)
PSA ^l^																				
Median (IQR) in ng/mL	6.8 (7.0)	7.0 (7.3)	5.8 (4.4)	7.5 (13.9)	7.4 (9.5)	6.8 (7.0)	6.9 (6.5)	6.1 (4.6)	7.5 (10.9)	6.8 (6.6)	6.8 (7.0)	6.8 (6.6)	6 (4.25)	7.4 (11.2)	6.6 (7.3)	6.8 (7.0)	6.5 (6.7)	5.8 (4.2)	7.6 (11.1)	6.7 (6.6)

^a^ Cases recruited within 2 years after disease diagnosis with an average interval between diagnosis and enrollment of 6.7 months; ^b^ AA: African American; ^c^ EA: European American; ^d^ Age at diagnosis; ^e^ IQR: Interquartile range; ^f^ SD: Standard deviation; ^g^ First-degree relative with prostate cancer; ^h^ Smoking status describes cigarette smoking; ^i^ Pathologically confirmed using *American Joint Committee on Cancer (AJCC) 7th Edition*; ^j^ Cases with pathologically confirmed T1 or T2 and Gleason score ≤ 7; ^k^ Cases with pathologically confirmed T3 or T4 or Gleason score > 7; ^l^ PSA: Prostate-specific antigen.

**Table 2:** Association of systemic indices with all-cause mortality in men with prostate cancer.

	All Cases			African American			European American			P Heterogeneity
NLR	Alive *N* (%)	Dead *N* (%)	* HR (95% CI)	Alive *N* (%)	Dead *N* (%)	* HR (95% CI)	Alive *N* (%)	Dead *N* (%)	* HR (95% CI)	
Low ≤ 2.9	228 (73)	97 (61)	Ref	142 (82)	68 (73)	Ref	86 (62)	29 (44)	Ref	
High > 2.9	84 (27)	62 (39)	1.34 (0.94–1.89)	32 (18)	25 (27)	1.33 (0.80–2.21)	52 (38)	37 (56)	1.27 (0.74–2.17)	
Continuous			1.23 (1.03–1.48)			1.26 (1.00–1.59)			1.25 (0.89–1.76)	
**PLR**										
Low ≤133.7	163 (52)	70 (44)	Ref	96 (55)	40 (43)	Ref	67 (49)	30 (45)	Ref	
High >133.7	149 (48)	88 (56)	1.10 (0.78–1.53)	78 (45)	52 (57)	1.48 (0.92–2.38)	71 (51)	36 (55)	0.88 (0.52–1.50)	
Continuous			1.13 (0.89–1.43)			1.33 (0.94–1.88)			1.14 (0.90–1.44)	
**SII**										
Low ≤ 430.8	142 (46)	51 (32)	Ref	98 (56)	34 (37)	Ref	44 (32)	17 (26)	Ref	
High > 430.8	170 (54)	107 (68)	1.47 (1.04–2.09)	76 (44)	58 (63)	1.66 (1.06–2.60)	94 (68)	49 (74)	1.07 (0.60–1.91)	0.01
Continuous			1.20 (1.02–1.41)			1.23 (0.99–1.52)			1.16 (0.88–1.54)	
**SIRI**										
Low ≤ 0.9	134 (43)	45 (28)	Ref	94 (54)	37 (40)	Ref	40 (29)	8 (12)	Ref	
High > 0.9	177 (57)	113 (72)	1.65 (1.14–2.41)	80 (46)	55 (60)	1.55 (0.99–2.43)	97 (71)	58 (88)	1.87 (0.83–4.22)	
Continuous			1.17 (1.02–1.33)			1.22 (1.02–1.46)			1.14 (0.90–1.44)	<0.01

* Unconditional Cox regression adjusted for age at study entry, self-reported race (not included in stratified analysis), BMI (kg/m^2^), aspirin use (no/yes), smoking history (never, former, current), diabetes (no/yes), education (high school or less, some college, college, professional school), family history of prostate cancer (first-degree relatives, no/yes), and treatment (0 = none, 1 = surgery, 2 = radiation, 3 = hormone, 4 = combination), disease stage (1 = stage I, 2 = stage IIA and IIB, 3 = stage III, 4 = stage IV), Gleason score (0 = Gleason ≤ 7 and 1 = Gleason > 7). CI = confidence interval; HR = hazard ratio.

**Table 3:** Association of systemic indices with prostate cancer-specific mortality in men with prostate cancer.

	All Cases			African American			European American			P Heterogeneity
NLR	Alive *N* (%)	Dead *N* (%)	* HR (95% CI)	Alive *N* (%)	Dead *N* (%)	* HR (95% CI)	Alive *N* (%)	Dead *N* (%)	* HR (95% CI)	
Low ≤ 2.9	305 (71)	20 (50)	Ref	194 (80)	16 (64)	Ref	111 (59)	4 (27)	Ref	
High > 2.9	126 (29)	20 (50)	2.56 (1.25–5.25)	48 (20)	9 (36)	2.96 (1.10–7.98)	78 (41)	11 (73)	2.43 (0.57–10.42)	<0.01
Continuous			1.51 (1.05–2.18)			1.40 (0.89–2.22)			1.84 (0.94–3.59)	
**PLR**										
Low ≤133.7	219 (51)	14 (35)	Ref	126 (52)	10 (40)	Ref	93 (49)	4 (27)	Ref	
High >133.7	211 (49)	26 (65)	1.55 (0.74–3.27)	115 (48)	15 (60)	1.75 (0.65–4.74)	96 (51)	11 (73)	1.87 (0.41–8.49)	
Continuous			1.11 (0.68–1.81)			0.89 (0.44–1.80)			1.47 (0.71–3.05)	
**SII**										
Low ≤430.8	184 (43)	9 (22)	Ref	124 (51)	8 (32)	Ref	60 (32)	1 (7)	Ref	
High >430.8	246 (57)	31 (78)	3.13 (1.37–7.16)	117 (49)	17 (68)	2.63 (1.0–6.97)	129 (68)	14 (93)	11.63 (1.03–131.02)	
Continuous			1.34 (0.96–1.87)			1.14 (0.77–1.68)			1.77 (1.00–3.13)	
**SIRI**										
Low ≤0.9	171 (40)	8 (21)	Ref	123 (51)	8 (33)	Ref	48 (26)		Ref	
High >0.9	259 (60)	31 (79)	3.24 (1.31–8.06)	119 (49)	16 (67)	3.19 (1.12–9.04)	140 (74)	15 (100)	Not enough cases	
Continuous			1.43 (1.11–1.84)			1.58 (1.11–2.25)			1.32 (0.84–2.08)	

* Unconditional Cox regression adjusted for age at study entry, self-reported race (not included in stratified analysis), BMI (kg/m^2^), aspirin use (no/yes), smoking history (never, former, current), diabetes (no/yes), education (high school or less, some college, college, professional school), family history of prostate cancer (first-degree relatives, no/yes), and treatment (0 = none, 1 = surgery, 2 = radiation, 3 = hormone, 4 = combination), disease stage (1 = stage I, 2 = stage IIA and IIB, 3 = stage III, 4 = stage IV), Gleason score (0 = Gleason ≤ 7 and 1 = Gleason > 7). CI = confidence interval; HR = hazard ratio.

## Data Availability

The data underlying this article will be shared upon reasonable request to the corresponding author.

## Supplementary Materials

The following supporting information can be downloaded at: https://www.mdpi.com/article/10.3390/cancers15061869/s1. Figure S1: Receiver operating characteristic (ROC) curves for cancer-specific survival related to the four systemic inflammation indices, NLR, PLR, SII, and SIRI. Table S1: ROC curve analysis related to the four systemic inflammation indices, NLR, PLR, SII, and SIRI. Table S2: Cases that are ineligible for inclusion in the study.
