# Supplementary material for: Systemic Inflammation Indices and Association with Prostate Cancer Survival in a Diverse Patient Cohort
Source: Cancers (Basel). 2023 Mar 20;15(6):1869. doi: 10.3390/cancers15061869 (PMC10047449; doi:10.3390/cancers15061869)
Supplement: Supplementary file 1 [file cancers-15-01869-s001.zip › cancers-2262120-supplementary.pdf]

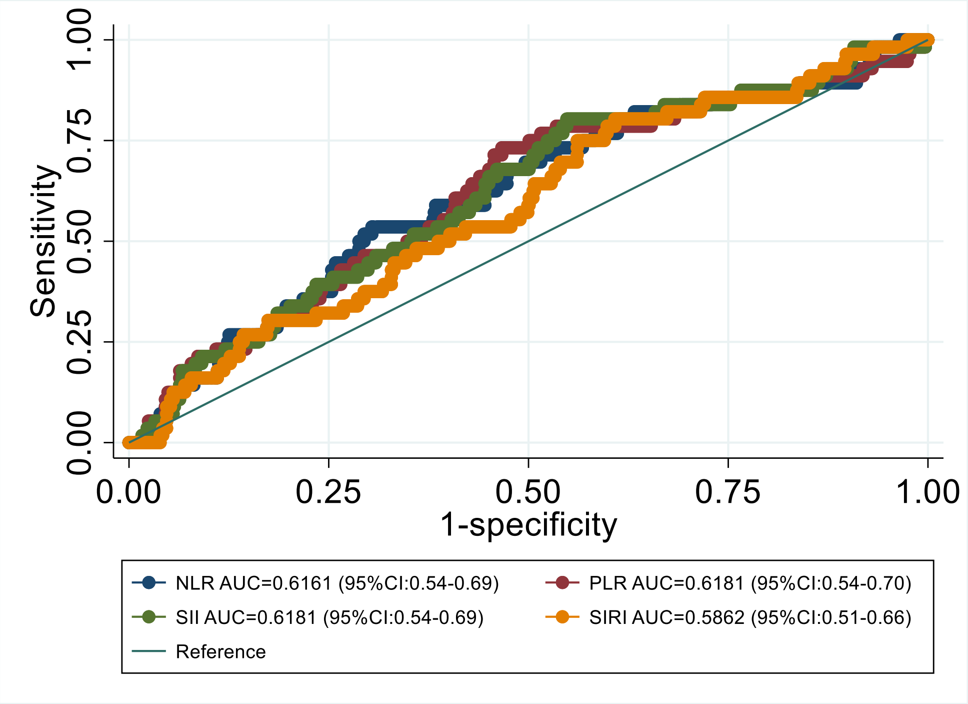

Supplementary Figure S1. Receiver operating characteristic (ROC) curves for cancer-specific survival related to the four systemic inflammation indices, NLR, PLR, SII and SIRI. Abbreviations: AUC = area under curve; CI = confidence interval.

Supplementary Table S1. ROC curve analysis related to the four systemic inflammation indices, NLR, PLR, SII and SIRI

	AUC	Lower CI	Upper CI	Sensitivity	Specificity	Cut-Off Value
NLR	0.6161	0.5382	0.6941	0.5	0.72	2.86
PLR	0.6181	0.5398	0.6964	0.7	0.54	133.66
SII	0.6181	0.5421	0.6942	0.79	0.45	430.8
SIRI	0.5862	0.5101	0.6623	0.78	0.4	0.9

Supplementary Table S2. Cases that are ineligible for inclusion in the study.

**Ineligible Cases[a]**			
**Demographics of Cases**	**All (n=374)**	**AA[b] (n=139)**	**EA[c] (n=235)**
Age[d]			
Median (IQR[e]) in years	65 (12)	62 (13)	66 (12)
BMI			
Mean (SD[f]) in kg/m^2	28.3 (4.7)	28.9 (5.4)	28.0 (5.3)
Education, *N(%)*			
High school or less	98 (26)	64 (46)	34 (14)
Some college	72 (19)	29 (21)	43 (18)
College	91 (24)	23 (16)	68 (29)
Graduate	95 (25)	12 (9)	83 (35)
Did not provide	20 (5)	12 (9)	8 (3)
**Baseline Health Factors**			

Family history of prostate cancer[g], *N (%)*			
No	325 (86)	118 (84)	207 (88)
Yes	30 (8)	9 (6)	21 (9)
Did not provide	21 (6)	13 (9)	8 (3)
Smoking status[h], *N (%)*			
Current	62 (16)	38 (27)	24 (10)
Former	150 (40)	53 (38)	97 (41)
Never	145 (39)	39 (28)	106 (45)
Did not provide	19 (5)	10 (7)	9 (4)
Stage[i], *N(%)*			
T1	79 (21)	16 (11)	63 (27)
T2	231 (61)	98 (70)	133 (56)
T3	45 (12)	15 (11)	30 (13)
T4	18 (5)	9 (6)	9 (4)
Missing	3 (1)	2 (1)	1 (<1)
Gleason score, *N (%)*			
≤7	328 (87)	122 (87)	206 (87)
>7	45 (12)	16 (11)	29 (12)
Missing	3 (<1)	2 (1)	1 (1)
Disease aggressiveness, *N (%)*			
Nonaggressive disease[J]	286 (76)	105 (75)	181 (77)
Aggressive disease[k]	87 (23)	33 (24)	54 (23)
Missing	3 (1)	2 (1)	1 (<1)
PSA			
Median (IQR) in ng/ml	5.8 (4.65)	6.7 (7)	5.7 (3.4)

[a]Cases with missing complete blood count data or cases with complete blood count taken more than 1 year post biopsy.

[b] AA: African-American

[c] EA: European American

[d] Age at diagnosis

[e] IQR: Interquartile range

[f] SD: Standard deviation

[g] First-degree relative with prostate cancer

[h] Smoking status describes cigarette smoking

[i] Pathologically confirmed using American Joint Committee on Cancer (AJCC) 7th Edition

[j] Cases with pathologically confirmed T1 or T2 and Gleason score ≤7

[k] Cases with pathologically confirmed T3 or T4 or Gleason score >7

[l] PSA: Prostate specific antigen
